# DNMT3B Knockdown Enhances PARP Inhibitor Sensitivity in Biliary Tract Cancer Cells via Opioid Growth Factor Receptor-Mediated Homologous Recombination Impairment

**DOI:** 10.3390/cancers17243936

**Published:** 2025-12-09

**Authors:** Soichiro Oda, Kazumichi Kawakubo, Masaki Kuwatani, Shugo Tanaka, Katsuma Nakajima, Shoya Shiratori, Hiroki Yonemura, Shunichiro Nozawa, Koji Hirata, Ryo Sugiura, Naoya Sakamoto

**Affiliations:** 1 Department of Gastroenterology and Hepatology, Hokkaido University Hospital, North 14, West 5, Kita-ku, Sapporo 060-8648, Japan; ode.930301@gmail.com (S.O.);; 2 Department of Pathology, Faculty of Medicine and Graduate School of Medicine, Hokkaido University, North 15, West 7, Kita-ku, Sapporo 060-8638, Japan

**Keywords:** biliary tract cancer, DNMT3B, OGFR, PARP inhibitor, homologous recombination, azacitidine, epigenetic modulation, synthetic lethality

## Abstract

**Background**: Biliary tract cancer (BTC) is an aggressive malignancy with poor prognosis and limited therapeutic options. Poly(ADP-ribose) polymerase (PARP) inhibitors have demonstrated efficacy in tumors with homologous recombination repair (HRR) deficiency. However, actionable BRCA1/2 mutations are rare in BTC. Epigenetic modulation via DNA methyltransferase (DNMT) inhibition is a proposed strategy for inducing an HR-deficient (“BRCAness”) phenotype and thereby enhancing therapeutic response to PARP inhibitors. This study aimed to determine whether the DNMT inhibitor azacitidine (AZA) enhances the antitumor effects of the PARP inhibitor niraparib (NIR) and to identify molecular mechanisms underlying this interaction. **Methods**: Two BTC cell lines, TFK-1 and RBE, were treated with AZA and/or NIR or subjected to siRNA-mediated DNMT1, DNMT3A, or DNMT3B knockdown. Functional analyses included homologous recombination (HR) assays, flow cytometric evaluation of cell-cycle distribution and apoptosis, proliferation and survival assays, and IC_50_ determination. Whole-transcriptome RNA sequencing was performed to identify differentially expressed genes after AZA treatment or DNMT3B knockdown, followed by validation via qPCR and Western blotting. To explore epigenetic regulation, whole-genome bisulfite sequencing was performed on TFK-1 cells following DNMT3B knockdown. **Results**: AZA treatment decreased HR frequency in a dose-dependent manner and enhanced the sensitivity of BTC cells to NIR, as evidenced by increased apoptosis, suppressed proliferation, and reduced IC_50_ values. DNMT3B knockdown recapitulated these effects, establishing a causal relationship between DNMT3B suppression and disrupted HR repair. RNA sequencing identified opioid growth factor receptor (OGFR) as a commonly upregulated gene after DNMT3B knockdown. Functional validation showed that OGFR overexpression reduced HR activity, increased apoptosis, and enhanced NIR sensitivity. Contrarily, OGFR knockdown conferred relative resistance. Whole-genome bisulfite sequencing showed no significant CpG methylation changes at the OGFR promoter region, indicating that OGFR induction is mediated through DNMT3B-dependent transcriptional regulation rather than direct promoter demethylation. **Conclusions**: DNMT3B inhibition sensitizes BTC cells to PARP inhibitors by disrupting HR repair. OGFR was identified as a novel regulator of HR and PARP inhibitor sensitivity, controlled via noncanonical DNMT3B-dependent transcriptional mechanisms that operate independently of CpG methylation. These findings provide new mechanistic insights into the epigenetic control of DNA repair and support the rationale for combining DNMT and PARP inhibitors as a promising therapeutic strategy for BTC beyond genetically HR-deficient cases.

## Simple Summary

Biliary tract cancer (BTC) is a rare but highly fatal malignancy with limited treatment options. This study shows that inhibition of DNMT3B enhances the sensitivity of BTC cells to the PARP inhibitor niraparib by disrupting homologous recombination repair via OGFR upregulation. These findings suggest that the combined use of DNMT and PARP inhibitors could represent a novel therapeutic strategy even for BTCs lacking BRCA mutations.

## 1. Introduction

Biliary tract cancer (BTC) is a relatively rare malignancy originating from biliary epithelial cells (cholangiocytes) lining the intrahepatic and extrahepatic bile ducts. However, its incidence has been increasing worldwide in recent years, particularly in East Asian countries [1,2]. Globally, approximately 210,000 new cases are diagnosed annually. In Japan, roughly 22,000 new cases and 18,000 deaths occur each year [3,4].

A major challenge in cholangiocarcinoma management is the high proportion of patients presenting with unresectable disease at diagnosis, resulting in limited treatment options and poor prognosis [5]. Even with surgical resection, recurrence is common, and the 5-year survival rates remain low, particularly in unresectable cases [6,7]. This highlights the need for more effective systemic therapies.

Despite recent advances in targeted therapies, such as those targeting FGFR2 fusion genes and immune checkpoint inhibitors, improvements in overall survival remain modest. FGFR2 gene fusions occur in approximately 10–15% of intrahepatic cholangiocarcinomas and have resulted in the approval of FGFR inhibitors, such as pemigatinib, infigratinib, and futibatinib [8]. Similarly, immune checkpoint blockade (e.g., PD-L1 inhibitors combined with gemcitabine and cisplatin) has demonstrated efficacy in recent clinical trials [9]. Nevertheless, the majority of patients lack these targetable alterations or fail to achieve durable benefit, indicating a persistent unmet clinical need.

Recently, poly(ADP-ribose) polymerase (PARP) inhibitors have emerged as promising treatments for tumors harboring homologous recombination repair (HRR) gene deficiencies [10,11,12]. In BTCs, germline or somatic mutations in HRR-related genes, including BRCA1/2, BAP1, ARID1A, and ATM, are detected in up to 30–60% of cases, although pathogenic BRCA1/2 mutations are relatively rare (approximately 1–7%) [13,14] The efficacy of PARP inhibitors in HR-deficient BTCs is currently being explored in clinical trials [15]. However, as only a subset of patients carry actionable HRR mutations, there is an urgent need for strategies that broaden the applicability of PARP inhibition by inducing or mimicking HRD and by creating complementary synthetic lethal contexts (e.g., combinations that increase replication stress or disrupt parallel DNA damage response pathways) [16].

Preclinical studies on other tumor types have reported that the combined use of DNA methylation and PARP inhibitors can enhance therapeutic efficacy. Mechanistic insights suggest that DNA methylation inhibition induces cell-cycle arrest and disrupts DNA-repair pathways, thereby sensitizing tumor cells to PARP blockade, although the precise mechanisms remain incompletely understood [17,18].

In this context, the present study aimed to determine whether DNA methyltransferase (DNMT) inhibition enhances the antitumor efficacy of PARP inhibitors in BTC cell lines in vitro. Furthermore, exploratory analyses were conducted to identify gene expression changes associated with altered therapeutic responses, aiming to elucidate potential mechanisms of synergy and identify candidate biomarkers [16].

## 2. Materials and Methods

### 2.1. Cell Lines and Cell Culture

The BTC cell lines TFK-1 and RBE were obtained from RIKEN BioResource Research Center (Kyoto, Japan). Cells were cultured in high-glucose Dulbecco’s Modified Eagle’s Medium (Gibco, Life Technologies, Cergy Pontoise, France) supplemented with 10% fetal bovine serum and 1% penicillin–streptomycin under standard conditions (37 °C, 5% CO_2_).

### 2.2. Reagents

5-Azacitidine (AZA; HY-10586) and niraparib (NIR; MK-4827) were purchased from MedChemExpress (Monmouth Junction, NJ, USA) and Selleckchem (Houston, TX, USA), respectively. Stock solutions (10^−2^ M) were prepared in sterile DMSO, aliquoted, stored at −20 °C, and freshly diluted in culture medium just before use.

The following small interfering RNAs (siRNAs) targeting DNMT genes were obtained from Qiagen (Courtaboeuf, France) and stored at −20 °C until use: siDNMT3B-3 (Qiagen Cat. No. SI04346668), siDNMT3B-10 (Qiagen Cat. No. SI04292673), siDNMT3A-11 (Qiagen Cat. No. SI04197437), siDNMT3A-15 (Qiagen Cat. No. SI04194654), siDNMT1-1 (Qiagen Cat. No. SI02654418), and siDNMT1-6 (Qiagen Cat. No. SI02654425).

A nontargeting control siRNA (siNTC; Qiagen Cat. No. 1027280) was used as a negative control.

Short hairpin RNAs (shRNAs) targeting the opioid growth factor receptor (OGFR) were purchased from Sigma-Aldrich (St. Louis, MO, USA) and corresponded to Clone IDs TRCN0000415480 and TRCN0000415278. A nontargeting control shRNA (shNTC; Sigma-Aldrich Cat. No. SHC016) was used as a control vector.

For forced OGFR overexpression, the cDNA corresponding to NM_007346 was used (VectorBuilder, Cat. No. VB010000-9492agg; St. Louis, MO, USA). An empty vector with the same backbone (VectorBuilder, Cat. No. VB010000-9492agg) lacking the OGFR insert served as the overexpression control. The antibody against OGFR used for Western blotting was obtained from Sigma-Aldrich (Cat. No. NP_0313722) and stored at −20 °C until use.

### 2.3. In Vitro AZA Treatment for TFK-1 and RBE

#### 2.3.1. Homologous Recombination Assay with AZA Treatment via qPCR

TFK-1 and RBE cells were seeded at a density of 1.0 × 10^6^ cells/35-mm dish and incubated for 24 h. After being washed with Phosphate-buffered saline (PBS), the culture medium was replaced and then added with 5-µM AZA; an equivalent dimethyl sulfoxide (DMSO) concentration was added to control wells. After 24 h, the cells were transfected with 0.5-µg/mL dl plasmid or 0.5-µg/mL positive-control plasmid (HR Assay Kit; NORGEN BioTek, Thorold, ON, Canada). After further incubation for 24 h, genomic DNA was extracted using the QIAamp DNA Kit (QIAGEN, Hilde, Germany). DNA samples were mixed with 2 µL of the Assay Primer Mixture (HR Assay Kit) and subjected to qPCR using a QuantStudio™ 3 Real-Time PCR Instrument (Thermo Fisher Scientific, Waltham, MA, USA).

qPCR was performed under the following conditions: initial denaturation at 95 °C for 3 min, followed by 40 cycles of denaturation at 95 °C for 15 s, annealing at 61 °C for 15 s, and extension at 72 °C for 15 s. Subsequently, melting curve analysis was conducted.

#### 2.3.2. Quantitative Analysis of Cell-Cycle Distribution After AZA Treatment via Flow Cytometry

Cells were seeded at a density of 1.0 × 10^6^ cells/35-mm dish. After incubation, cells were washed with PBS, the medium was replaced, and 5-µM AZA was added.; an equivalent DMSO concentration was added to control wells. After 24-h incubation, cells were harvested, resuspended in PBS, and stained with 0.05-mg/mL Cell-Cycle Assay Solution (DOJINDO, Mashiki, Japan) at 37 °C for 15 min.

Flow cytometric analysis was conducted using a FACSVerse flow cytometer (BD Biosciences, Franklin Lakes, NJ, USA) with an excitation filter at 633–647 nm and a fluorescence emission filter at 695 nm. Data acquisition and analysis were conducted using BD FACSuite (BD Biosciences, Franklin Lakes, NJ, USA).

The concentrations of AZA and NIR used in this study were selected based on preliminary dose–response assessments. For NIR, both TFK-1 and RBE cells began to exhibit a decline in cell viability at approximately 5 µM (Appendix A). The IC_50_ values of NIR for TFK-1 and RBE were 9.7 μM (7.9–11.9 μM) and 30.8 μM (4.2–227.7 μM), respectively. Because the primary aim of this study was to investigate strategies to overcome PARP inhibitor resistance, we selected a concentration at which PARP inhibitor unresponsiveness was preserved. For AZA, 5 µM resulted in the most pronounced G2/M accumulation among the tested conditions in the cell-cycle analyses; therefore, this concentration was adopted for subsequent experiments.

#### 2.3.3. Quantitative Analysis of Cell Death After AZA and NIR Treatments via Flow Cytometry

BTC cells were seeded at a density of 1.0 × 10^6^ cells/35-mm dish and incubated for 24 h. After PBS washing and medium replacement, cells were treated with 5 µM AZA and/or 5 µM NIR as indicated. After further incubation for 48 h, cells were harvested, resuspended in PBS, and stained with 0.05-mg/mL propidium iodide (Nacalai Tesque, Kyoto, Japan) at room temperature (20–25 °C) for 15 min.

Dead cells were detected via flow cytometry using an excitation filter at 480–550 nm and a fluorescence emission filter at 590 nm. Data acquisition and analysis were conducted, as described above.

#### 2.3.4. Quantitative Evaluation of Cell Proliferation After AZA and NIR Treatments via Spectrophotometry

BTC cells were seeded at a density of 1.0 × 10^6^ cells/35-mm dish. After 24 h incubation, 5-µM AZA and/or 5-µM NIR were added.

Cell viability was evaluated using Cell Counting Kit-8 (DOJINDO, Mashiki, Japan), and absorbance was measured at 450 nm using a microplate spectrophotometer (SpectraMax iD3; WAKENYAKU, Kyoto, Japan). Measurements were taken every 24 h from days 0 to 4.

#### 2.3.5. Quantitative Evaluation of Cell Survival After AZA and NIR Treatments via Spectrophotometry

BTC cells were seeded at a density of 1.0 × 10^6^ cells/35-mm dish. After incubation, 5-µM AZA was added. After further incubation for 24 h, cells were harvested, resuspended in PBS, and reseeded at a density of 1.0 × 10^4^ cells/well in a 96-well plate. After further incubation for 24 h, NIR was added at various concentrations (0, 0.2, 0.5, 1.0, 2.0, 5.0, 10.0, 20.0, and 50.0 µM), whereas control wells received corresponding DMSO concentrations.

After further incubation for 24 h, cell viability was evaluated using Cell Counting Kit-8 (DOJINDO, Japan), and absorbance was measured at 450 nm using a microplate spectrophotometer (SpectraMax iD3; WAKENYAKU, Kyoto, Japan). Data were analyzed using a four-parameter logistic regression model to determine IC_50_ values and their 95% confidence intervals (CIs).

### 2.4. Knockdown of DNMT Genes

BTC cells were seeded at 1.0 × 10^6^ cells/35 mm. After incubation, the medium lacking 1% penicillin–streptomycin was replaced. A transfection mixture containing 9 µL of Lipofectamine™ RNAiMAX Reagent and 3 µL of 10-µM siRNA targeting DNMT genes was prepared in 150-µL Opti-MEM™ Reduced Serum Medium (Thermo Fisher Scientific). After 5 min, the mixture was sdded dropwise at room temperature. Knockdown efficiency for each target DNMT gene was confirmed via qPCR.

#### 2.4.1. Homologous Recombination Assay After DNMT Knockdown via qPCR

Cells were seeded at a density of 1.0 × 10^6^ cells and incubated for 24 h. Each DNMT was knocked down, as described above, and control cells were transfected with siNTC. After 24 h, cells were transfected with either 0.5-µg/mL dl plasmid or 0.5-µg/mL positive-control plasmid (HR Assay Kit; NORGEN BioTek, Canada). After further incubation for 24 h, genomic DNA was extracted using the QIAamp DNA Kit (QIAGEN, Germany). DNA samples were mixed with 2 µL of the Assay Primer Mixture and subjected to qPCR using a QuantStudio™ 3 Real-Time PCR Instrument (Thermo Fisher Scientific, Waltham, MA, USA).

qPCR was performed under the following conditions: initial denaturation at 95 °C for 3 min, followed by 40 cycles of denaturation at 95 °C for 15 s, annealing at 61 °C for 15 s, and extension at 72 °C for 15 s. Subsequently, melting curve analysis was conducted.

#### 2.4.2. Quantitative Analysis of Cell-Cycle Distribution After DNMT Knockdown

TFK-1 and RBE cells were seeded at a density of 1.0 × 10^6^ cells/35-mm dish and incubated for 24 h. Each DNMT was knocked down, as described above, and control cells were transfected with siNTC. After 48-h incubation, cells were harvested, resuspended in PBS, and stained with 0.05-mg/mL Cell-Cycle Assay Solution (DOJINDO, Japan) at 37 °C for 15 min.

Flow cytometric analysis was conducted using a FACSVerse flow cytometer (BD Biosciences, Franklin Lakes, NJ, USA) with an excitation filter at 633–647 nm and a fluorescence emission filter at 695 nm. Data acquisition and analysis were conducted using BD FACSuite.

#### 2.4.3. Quantitative Analysis of Cell Death After DNMT Knockdown and NIR Treatment

TFK-1 and RBE cells were seeded at a density of 1.0 × 10^6^ cells/35-mm dish and incubated for 24 h. Each DNMT was knocked down, as described above, and control cells were transfected with siNTC. After 24 h, the cells were washed with PBS, and then medium was replaced and added with 5-µM NIR; control wells received the same DMSO concentration. After further incubation for 48 h, cells were harvested, resuspended in PBS, and stained with 0.05-mg/mL propidium iodide (Nacalai Tesque, Japan) at room temperature (20–25 °C) for 15 min.

Dead cells were detected via flow cytometry. Data acquisition and analysis were conducted, as described above.

#### 2.4.4. Quantitative Evaluation of Cell Proliferation After DNMT Knockdown and NIR Treatment via Spectrophotometry

TFK-1 and RBE cells were seeded at a density of 1.0 × 10^6^ cells/35-mm dish and incubated for 24 h. Each DNMT was knocked down, as described above, and control cells were transfected with siNTC. After 24 h, cells were harvested, resuspended in PBS, and reseeded at a density of 1.0 × 10^4^ cells/well in a 96-well plate. After further incubation for 24 h, 5-µM NIR was added, whereas control wells received the same DMSO concentration.

Cell viability was evaluated using Cell Counting Kit-8 (DOJINDO, Japan), and absorbance was measured at 450 nm using a microplate spectrophotometer (SpectraMax iD3; WAKENYAKU, Kyoto, Japan). Measurements were taken every 24 h from days 0 to 4.

#### 2.4.5. Quantitative Evaluation of Cell Survival After DNMT Knockdown and NIR Treatment via Spectrophotometry

TFK-1 and RBE cells were seeded at a density of 1.0 × 10^6^ cells/35-mm dish and incubated for 24 h. Each DNMT was knocked down, as described above, and control cells were transfected with siNTC. After 24 h, cells were harvested, resuspended in PBS, and reseeded at a density of 1.0 × 10^4^ cells/well in a 96-well plate. After further incubation for 24 h, NIR was added at various concentrations (0, 0.2, 0.5, 1.0, 2.0, 5.0, 10.0, 20.0, and 50.0 µM), whereas control wells received corresponding DMSO concentrations.

After further incubation for 24 h, cell viability was evaluated using Cell Counting Kit-8 (DOJINDO, Japan), and absorbance was measured at 450 nm using a microplate spectrophotometer (SpectraMax iD3; WAKENYAKU, Kyoto, Japan). Data were analyzed using a four-parameter logistic regression model to determine the IC_50_ values and their 95% CIs.

### 2.5. RNA Sequencing After AZA Treatment or DNMT3B Knockdown

TFK-1 and RBE cells were treated with AZA or subjected to DNMT3B knockdown (3B-3 and 3B-10), as described above. The controls included DMSO-treated cells for AZA experiments and nontargeting siNTC for DNMT3B experiments.

Total RNA was extracted using the RNeasy Mini Kit (QIAGEN, Germany). High-quality RNA samples were sent to Rhelixa Inc. (Tokyo, Japan) for whole-genome RNA sequencing. Poly(A)+ RNA was purified using the NEBNext Poly(A) mRNA Magnetic Isolation Module (New England Biolabs, Ipswich, MA, USA), and sequencing libraries were prepared using the NEBNext Ultra II Directional RNA Library Prep Kit (New England Biolabs). Paired-end sequencing (150 bp × 2) was performed on an Illumina NovaSeq X Plus platform (Illumina, San Diego, CA, USA).

The obtained reads were mapped to the human reference genome (GRCh38) using a STAR aligner. Differentially expressed gene (DEG) analysis was conducted using the edgeR (ver 3.26.8.) software package, in which read counts were normalized using the Trimmed Mean of the M-values (TMM) method, and log_2_ fold change (log_2_FC) values were calculated [19,20]. Genes exhibiting statistically significant differences in log_2_FC between samples were identified as DEGs, and false discovery rate (FDR) values were computed to adjust for multiple testing [21].

### 2.6. Forced Expression and Knockdown of OGFR

Forced overexpression and knockdown of the OGFR gene in TFK-1 cells were achieved using lentiviral vectors. For overexpression, cells were transduced with a lentiviral vector carrying full-length human OGFR cDNA (VectorBuilder, Kanagawa, Japan) according to the manufacturer’s protocol. Transduction efficiency was improved by adding 5-µg/mL polybrene, and stable overexpression cell lines were generated via puromycin selection (0.5 µg/mL) for 7 days. Overexpression was confirmed via qPCR and Western blotting.

For knockdown, cells were transduced with shRNA lentiviral particles targeting OGFR. After 48 h, puromycin (0.5 µg/mL) selection was applied for 5–7 days, and successful knockdown was confirmed via qPCR and Western blotting.

Western blot band intensities were quantified using densitometry. For each blot, the signal intensities of the target protein and the corresponding loading control (β-actin) were measured using ImageJ software ver 1.54m (NIH). A rectangular region of interest (ROI) of identical size was applied to all lanes, and background subtraction was performed using the rolling-ball algorithm. The relative protein expression level was calculated as the ratio of target protein intensity to loading-control intensity (T/C). For each experiment, values were normalized to the control group, which was set to 1.0.

#### 2.6.1. Homologous Recombination Assay Using OGFR-Overexpressing/Knockdown TFK-1 Cells via qPCR

TFK-1 cells exhibiting OGFR overexpression/knockdown or control cells were seeded at a density of 1.0 × 10^6^ cells/35-mm dish and incubated for 24 h. Then, cells were transfected with 0.5-µg/mL dl plasmid or 0.5-µg/mL positive-control plasmid (HR Assay Kit; NORGEN BioTek, Canada). After further incubation for 24 h, genomic DNA was extracted using the QIAamp DNA Kit (QIAGEN, Germany). DNA samples were mixed with 2 µL of the Assay Primer Mixture (HR Assay Kit) and subjected to qPCR using a QuantStudio™ 3 Real-Time PCR Instrument (Thermo Fisher Scientific, Waltham, MA, USA).

qPCR was performed under the following conditions: initial denaturation at 95 °C for 3 min, followed by 40 cycles of denaturation at 95 °C for 15 s, annealing at 61 °C for 15 s, and extension at 72 °C for 15 s. Subsequently, melting curve analysis was conducted.

#### 2.6.2. Quantitative Analysis of Cell-Cycle Distribution in OGFR-Overexpressing/Knockdown TFK-1 Cells via Flow Cytometry

TFK-1 cells exhibiting OGFR overexpression/knockdown or control cells were seeded at a density of 1.0 × 10^6^ cells/35-mm dish and incubated for 24 h. After 48-h incubation, cells were harvested, resuspended in PBS, and stained with 0.05-mg/mL Cell-Cycle Assay Solution (DOJINDO, Japan) at 37 °C for 15 min.

Flow cytometric analysis was conducted using a FACSVerse flow cytometer (BD Biosciences, Franklin Lakes, NJ, USA) with an excitation filter at 633–647 nm and a fluorescence emission filter at 695 nm. Data acquisition and analysis were conducted using BD FACSuite (BD Biosciences).

#### 2.6.3. Quantitative Analysis of Cell Death in OGFR-Overexpressing/Knockdown TFK-1 Cells with NIR Treatment via Flow Cytometry

TFK-1 cells exhibiting OGFR overexpression/knockdown or control cells were seeded at a density of 1.0 × 10^6^ cells. After 24 h incubation, 5-µM NIR were added. Control wells received an equivalent DMSO concentration. After further incubation for 48 h, cells were harvested, resuspended in PBS, and stained with 0.05-mg/mL propidium iodide (Nacalai Tesque, Japan) at room temperature (20–25 °C) for 15 min.

Dead cells were detected via flow cytometry. Data acquisition and analysis were conducted, as described above.

#### 2.6.4. Quantitative Evaluation of Cell Proliferation in OGFR-Overexpressing/Knockdown TFK-1 Cells with NIR Treatment via Spectrophotometry

OGFR overexpression/knockdown TFK-1 cells were seeded at a density of 1.0 × 10^6^ cells. After incubation, cells were reseeded at a density of 1.0 × 10^4^ cells/well in a 96-well plate. After further incubation for 24 h, 5-µM NIR was added, whereas control wells received an equivalent DMSO concentration.

Cell viability was evaluated using Cell Counting Kit-8 (DOJINDO, Japan), using a microplate spectrophotometer (SpectraMax iD3; WAKENYAKU, Kyoto, Japan). Measurements were taken every 24 h from days 0 to 4.

#### 2.6.5. Quantitative Evaluation of Cell Survival in OGFR-Overexpressing/Knockdown TFK-1 Cells with NIR Treatment via Spectrophotometry

OGFR overexpression/knockdown TFK-1 cells were seeded at a density of 1.0 × 10^6^ cells. After incubation, cells were reseeded at a density of 1.0 × 10^4^ cells. After further incubation for 24 h, NIR was added at various concentrations (0, 0.2, 0.5, 1.0, 2.0, 5.0, 10.0, 20.0, and 50.0 µM), whereas control wells received corresponding DMSO concentrations.

After further incubation for 24 h, cell viability was evaluated using Cell Counting Kit-8 (DOJINDO, Japan), and absorbance was measured at 450 nm using a microplate spectrophotometer (SpectraMax iD3; WAKENYAKU, Kyoto, Japan).

### 2.7. Whole-Genome Bisulfite Sequencing After DNMT3B Knockdown

To elucidate the mechanism underlying OGFR upregulation after DNMT3B knockdown, whole-genome bisulfite sequencing was performed using DNA extracted from TFK-1 cells transfected with siDNMT3B-3, siDNMT3B-10, or siNTC.

Genomic DNA was extracted using the QIAamp DNA Kit (QIAGEN, Germany) and submitted to Rhelixa, Inc. (Tokyo, Japan), for whole-genome bisulfite sequencing. Library preparation was performed using standard bisulfite conversion and sequencing procedures. Furthermore, paired-end sequencing (150 bp × 2) was carried out on an Illumina NovaSeq X Plus platform (Illumina, San Diego, CA, USA).

Bioinformatics analysis was conducted by Rhelixa Inc. using the methylKit (ver 1.10.0) software package [22]. Read counts were normalized, and differentially methylated cytosines (DMCs) and differentially methylated regions (DMRs) were identified. The genome was divided into 1000-base-pair bins, and the methylation ratios for each DMC/DMR were calculated and mapped.

Based on the DMC/DMR profiles, DEG analysis was conducted using the edgeR package (Version 3.26.8) [20,23]. Read counts derived from DMC/DMRs were normalized using the TMM method, and the log_2_FC values were calculated. Genes exhibiting statistically significant differences in log_2_FC values between samples were extracted, and multiple testing correction was performed using the Benjamini–Hochberg method to calculate the FDR [23].

### 2.8. Statistical Analysis

Data are expressed as mean ± standard error of the mean (SEM) from at least three independent experiments (*n* > 3). Statistical analyses were conducted using EZR version 1.63 (Saitama Medical Center, Jichi Medical University), a graphical interface for R (R Foundation for Statistical Computing, Vienna, Austria). 

For multiple comparisons, one-way analysis of variance followed by Dunnett’s post hoc test was employed. A *p*-value < 0.05 was considered statistically significant.

## 3. Results

### 3.1. In Vitro AZA Treatment for TFK-1 and RBE

To explore the effects of AZA on cholangiocarcinoma cell lines, a series of in vitro experiments were conducted to compare control and AZA-treated groups.

#### 3.1.1. Homologous Recombination (HR) Assay with AZA Treatment via qPCR

**Figure 1:**
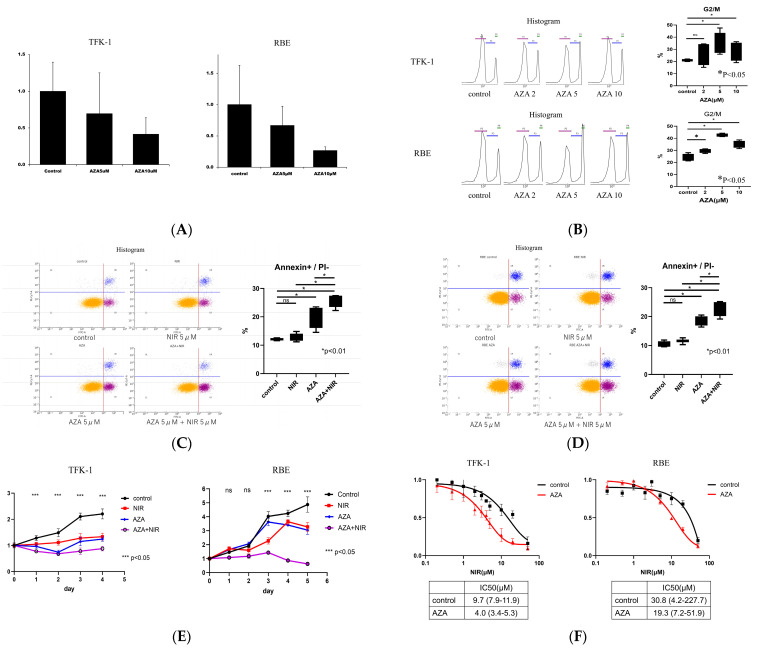
Effects of AZA and NIR treatments on BTC cells (TFK-1 and RBE). (**A**) Homologous recombination (HR) frequency evaluated via qPCR after AZA treatment. The HR frequency in the control group was set to 1. (**B**) Representative flow cytometric histograms and quantification of cell-cycle distribution following AZA treatment. The proportion of cells in the G2/M phase increased in a dose-dependent manner. (**C**,**D**) Flow cytometric analysis of apoptosis in TFK-1 (**C**) and RBE (**D**) cells following AZA and/or NIR treatment. Apoptotic cells were defined as Annexin V^+^/PI^−^. (**E**) Temporal changes in cell proliferation measured via CCK-8 assay following AZA and/or NIR treatment (days 0–4). Values were normalized to day 0. (**F**) Dose–response curves and IC_50_ values for NIR with or without AZA pretreatment, evaluated via CCK-8 assay. * Data are expressed as mean ± SEM. Statistical significance was determined via one-way ANOVA, followed by Dunnett’s post hoc test. Abbreviations: AZA, 5-azacitidine; NIR, niraparib; HR, homologous recombination; BTC, biliary tract cancer; PI, propidium iodide; SEM, standard error of the mean; qPCR, quantitative polymerase chain reaction.

To evaluate HR frequency in the control and AZA-treated groups, HR events were quantified using a qPCR-based HR Assay Kit. In TFK-1 cells, AZA treatment reduced HR frequency in a dose-dependent manner, with 5- and 10-μM AZA reducing HR activity to approximately 0.6 and 0.4 of control levels, respectively. A similar trend was observed in RBE cells, suggesting that AZA suppresses HR repair capacity in both cell lines (Figure 1A).

#### 3.1.2. Quantitative Analysis of Cell-Cycle Distribution After AZA Treatment via Flow Cytometry

To evaluate the effects of AZA on cell-cycle distribution, TFK-1 and RBE cells were treated with AZA at concentrations of 2, 5, and 10 μM. Cell-cycle profiles were analyzed using a cell-cycle assay solution and quantified via flow cytometry. In both cell lines, AZA treatment caused a concentration-dependent increase in the proportion of cells in the G2/M phase. Representative flow cytometric histograms and the corresponding quantification of G2/M-phase populations are presented in Figure 1B.

AZA typically induces a dose-dependent accumulation of cells in the G2/M phase [24]. However, in our study, the proportion of G2/M-phase cells at 10 µM AZA was slightly lower than that at 5 µM. Similar findings have been reported in other cancer models, in which high-dose AZA induces substantial cell death, and dead or dying cells are frequently recognized as G1-like events during flow cytometric analysis [25]. This phenomenon can lead to an apparent relative decrease in the G2/M fraction. A comparable mechanism likely accounts for the mild reduction observed in our study.

#### 3.1.3. Quantitative Analysis of Cell Death After AZA and NIR Treatment via Flow Cytometry

To assess the increase in apoptotic cells induced by AZA and NIR treatments, TFK-1 and RBE cells were divided into four groups: control, NIR (5 μM), AZA (5 μM), and AZA (5 μM) + NIR (5 μM) treatment. Apoptosis was evaluated via dual staining with Annexin V and propidium iodide (PI), followed by flow cytometric analysis. Cells positive for Annexin V but negative for PI were classified as apoptotic cells, and their proportion relative to the total cell population was measured.

Compared with either NIR or AZA monotherapy, the AZA + NIR treatment increased the proportion of apoptotic cells in TFK-1 and RBE cells.

Representative FCM dot plots and the graphical presentation of the proportion of apoptotic cells are shown in Figure 1C for TFK-1 and Figure 1D for RBE.

#### 3.1.4. Quantitative Evaluation of Cell Proliferation After AZA and NIR Treatments via Spectrophotometry

To evaluate the temporal changes in cell proliferation induced by NIR (5 μM) in the control and AZA (5 μM)-treated groups, cell viability was assessed via CCK-8 assay from days 0 to 4. The cells were divided into four groups: control, AZA (5 μM), NIR (5 μM), and AZA (5 μM) + NIR (5 μM) treatment. The relative proliferation rate of each group was normalized to the mean absorbance value at day 0, which was set to 1.

For TFK-1 cells, the AZA + NIR group showed a significant reduction in cell proliferation compared with either AZA or NIR monotherapy at all time points from days 1 to 4.

For RBE cells, the AZA + NIR treatment significantly reduced cell proliferation compared with either monotherapy from day 2 onward (Figure 1E).

#### 3.1.5. Quantitative Evaluation of Cell Survival After AZA and NIR Treatments via Spectrophotometry

To further determine whether AZA pretreatment enhances the sensitivity of BTC cells to NIR, dose–response analyses were conducted via CCK-8 assay following exposure to increasing NIR concentrations (0–50 μM). IC_50_ values were calculated via nonlinear regression.

In TFK-1 cells, the IC_50_ of NIR was 9.7 μM (95% CI, 7.9–11.9 μM) in the control group, which markedly decreased to 4.0 μM (95% CI, 3.4–5.3 μM) after AZA pretreatment (5 μg/mL). Similarly, in RBE cells, the IC_50_ for NIR was 30.8 μM (95% CI, 4.2–227.7 μM) in the control group, which markedly decreased to 19.3 μM (95% CI, 7.2–51.9 μM) after the same pretreatment. Although the CIs were wide in RBE cells, a trend toward enhanced NIR sensitivity was observed after AZA treatment. These results indicated that AZA pretreatment potentiates the cytotoxic effect of NIR in BTC cells, particularly in TFK-1 cells (Figure 1F).

### 3.2. Knockdown of DNMT Genes

**Figure 2:**
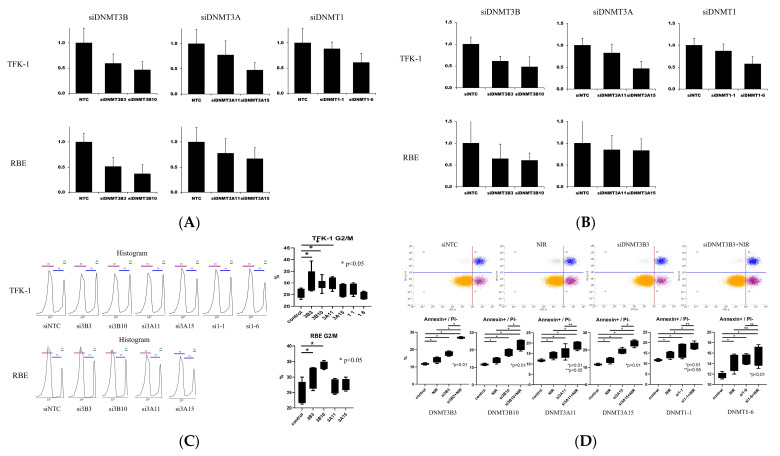

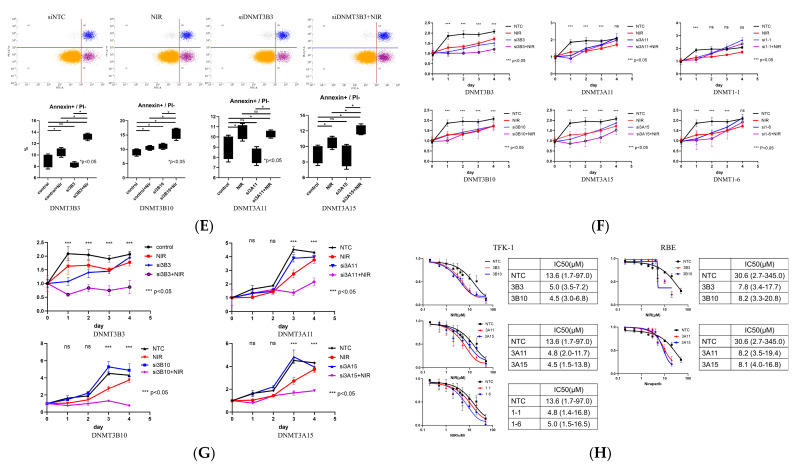
Effects of DNMT knockdown on HR activity, cell-cycle distribution, apoptosis, proliferation, and NIR sensitivity. (**A**) Validation of DNMT knockdown efficiency via qPCR in TFK-1 and RBE cells. Expression levels are shown relative to siNTC. (**B**) HR frequency quantified via qPCR following DNMT knockdown. Values were normalized to siNTC controls. (**C**) Representative flow cytometric histograms and quantification of cell-cycle distribution after DNMT knockdown. Increased G2/M-phase accumulation was observed, particularly after DNMT3B knockdown. (**D**,**E**) Flow cytometric analysis of apoptosis in TFK-1 (**D**) and RBE (**E**) cells after DNMT knockdown with or without NIR treatment. (**F**,**G**) Temporal changes in cell proliferation measured via CCK-8 assay after DNMT knockdown with or without NIR treatment in TFK-1 (**F**) and RBE (**G**) cells. (**H**) Dose–response curves and IC_50_ values for NIR after DNMT knockdown, evaluated via CCK-8 assay. * Data are expressed as mean ± SEM. Statistical significance was determined via one-way ANOVA, followed by Dunnett’s post hoc test. *p* < 0.05 compared with siNTC controls. Abbreviations: DNMT, DNA methyltransferase; siNTC, nontargeting siRNA control; HR, homologous recombination; PI, propidium iodide; NIR, niraparib; SEM, standard error of the mean; qPCR, quantitative polymerase chain reaction.

To validate the efficiency of siRNA-mediated knockdown of DNMT family members, TFK-1 and RBE cells were transfected with specific siRNAs, and mRNA expression was quantified via qPCR after 48 h relative to siNTC controls. Both siDNMT3B/3A/1 in TFK-1 cells and siDNMT3B/3A in RBE cells effectively reduced the expression levels of their respective target RNAs (Figure 2A).

#### 3.2.1. Homologous Recombination Assay After DNMT Knockdown via qPCR

To assess the effect of DNMT knockdown on HR repair, HR frequency was quantified via qPCR after siRNA transfection. DNMT3B and DNMT3A knockdown markedly reduced HR activity in TFK-1 and RBE cells, whereas DNMT1 knockdown exerted a more modest effect (Figure 2B).

#### 3.2.2. Quantitative Analysis of Cell-Cycle Distribution After DNMT Knockdown

To determine whether DNMT knockdown affected cell-cycle progression, flow cytometric analysis was conducted to measure the proportion of cells in the G2/M phase. DNMT3B knockdown, and to a lesser extent DNMT3A knockdown, increased the G2/M-phase population in TFK-1 and RBE cells, indicating impaired cell-cycle progression (Figure 2C).

#### 3.2.3. Quantitative Analysis of Cell Death After DNMT Knockdown and NIR Treatment

To assess the effect of DNMT knockdown on apoptosis under NIR treatment, TFK-1 and RBE cells transfected with siNTC or DNMT-targeting siRNAs were treated with DMSO or NIR (5 µM), followed by Annexin V/PI staining and flow-cytometric analysis. DNMT3B knockdown considerably increased the proportion of apoptotic cells, particularly in combination with NIR treatment, whereas DNMT3A knockdown exerted a moderate effect and DNMT1 knockdown showed minimal impact (Figure 2D for TFK-1, Figure 2E for RBE).

#### 3.2.4. Quantitative Evaluation of Cell Proliferation After DNMT Knockdown and NIR Treatment via Spectrophotometry

To evaluate the impact of DNMT knockdown on cell proliferation during NIR treatment, TFK-1 and RBE cells transfected with siNTC or DNMT-targeting siRNAs were cultured in the presence or absence of NIR (5 µM), and cell viability was assessed via CCK-8 assay from days 0 to 4. DNMT3B knockdown markedly suppressed cell proliferation and enhanced the antiproliferative effect of NIR in both cell lines, DNMT3A knockdown demonstrated moderate enhancement, and DNMT1 knockdown exhibited minimal or no synergistic effect (Figure 2F for TFK-1, Figure 2G for RBE).

#### 3.2.5. Quantitative Evaluation of Cell Survival After DNMT Knockdown and NIR Treatment via Spectrophotometry

To determine whether DNMT knockdown enhanced the sensitivity of BTC cells to NIR, dose–response analyses were conducted via CCK-8 assay across increasing NIR concentrations, and IC_50_ values were calculated via nonlinear regression. Knockdown of DNMT, particularly DNMT3B, markedly decreased the IC_50_ values in TFK-1 and RBE cells, indicating enhanced sensitivity to NIR (Figure 2H).

### 3.3. RNA Sequencing After AZA Treatment or DNMT3B Knockdown

**Figure 3:**
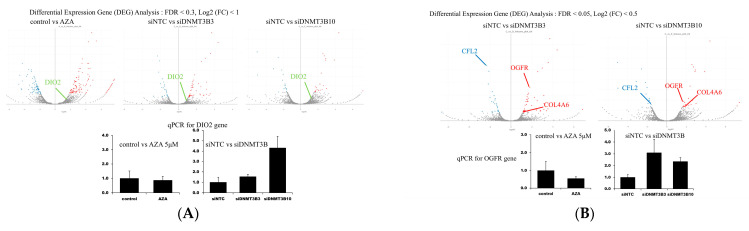
RNA sequencing analysis after AZA treatment or DNMT3B knockdown in TFK-1 cells. (**A**) Differentially expressed genes (DEGs) with FDR < 0.3, highlighting DIO2. qPCR validation for DIO2 is illustrated. (**B**) DEGs with FDR < 0.05, highlighting OGFR and others. qPCR validation confirmed OGFR upregulation after DNMT3B knockdown. * Statistical significance was determined via one-way ANOVA, followed by Dunnett’s post hoc test. *p* < 0.05 compared with control groups. Abbreviations: DEG, differentially expressed gene; FDR, false discovery rate; DIO2, deiodinase, iodothyronine type II; AZA, 5-azacitidine; DNMT, DNA methyltransferase; OGFR, opioid growth factor receptor; qPCR, quantitative polymerase chain reaction; SEM, standard error of the mean.

RNA sequencing and differential expression analysis revealed that 216 genes were significantly altered in the AZA-treated group (FDR < 0.05), whereas 70 and 44 genes were significantly altered in the siDNMT3B-3 and siDNMT3B-10 groups, respectively. Although no significant overlap was observed between the AZA-treated and siDNMT3B groups, five common DEGs were shared between siDNMT3B-3 and siDNMT3B-10, with OGFR being consistently upregulated. Owing to its reported role in cell-cycle regulation, OGFR was chosen for subsequent functional analyses. When the threshold was adjusted to q < 0.3, DIO2 appeared as a commonly altered gene across all groups; however, qPCR validation failed to confirm this change. Owing to the small number of significant DEGs, pathway enrichment analysis was not feasible (Figure 3A for DIO2, Figure 3B for OGFR validation).

### 3.4. Forced Expression and Knockdown of OGFR

**Figure 4:**
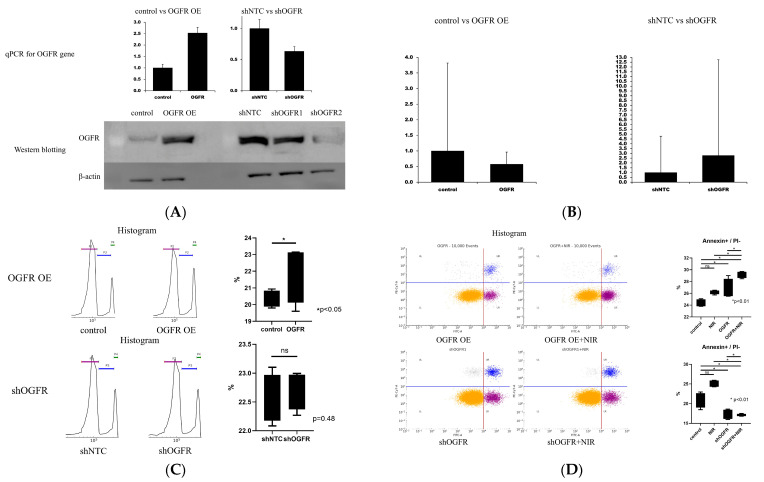

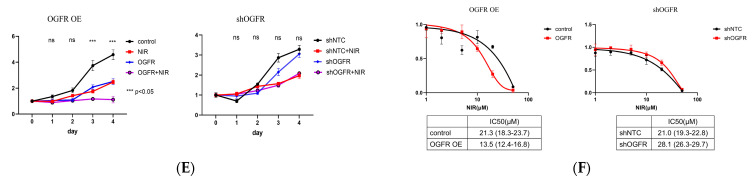
Functional analysis of OGFR in TFK-1 cells. (**A**) Validation of OGFR overexpression and knockdown via qPCR and Western blotting. shOGFR#1 and shOGFR#2 were generated using lentiviral vectors corresponding to Clone IDs TRCN0000415480 and TRCN0000415278, respectively (Sigma-Aldrich, St. Louis, MO, USA). (**B**) HR frequency evaluated via qPCR in OGFR-overexpressing and knockdown cells. OGFR overexpression reduced HR activity, whereas knockdown increased HR. (**C**) Representative flow cytometric histograms and quantification of cell-cycle distribution. OGFR overexpression induced a modest increase in the G2/M fraction, whereas knockdown exerted minimal effect. (**D**) Flow cytometric analysis of apoptosis in OGFR-overexpressing and knockdown cells with or without NIR treatment. OGFR overexpression enhanced apoptosis, particularly after NIR treatment. (**E**) Temporal changes in cell proliferation evaluated via CCK-8 assay in OGFR-overexpressing and knockdown cells with or without NIR treatment. OGFR overexpression suppressed proliferation after NIR treatment. (**F**) Dose–response curves and IC_50_ values for NIR in OGFR-overexpressing and knockdown cells. OGFR overexpression enhanced sensitivity to NIR, whereas knockdown conferred relative resistance. Abbreviations: OGFR, opioid growth factor receptor; OE, overexpression; shOGFR, short hairpin RNA–mediated knockdown of OGFR; HR, homologous recombination; NIR, niraparib; PI, propidium iodide; qPCR, quantitative polymerase chain reaction; SEM, standard error of the mean.

As RNA sequencing identified OGFR as a commonly upregulated gene in siDNMT3B-3 and siDNMT3B-10 cells, its functional role was investigated by establishing TFK-1 cell lines with OGFR forced overexpression or shRNA-mediated knockdown. As TFK-1 cells are highly sensitive to antibiotic selection, standard puromycin concentrations (1–2 μg/mL) induced extensive cell death and insufficient colony formation. Thus, a reduced concentration of 0.5 μg/mL was applied for both models. qPCR analysis confirmed successful OGFR expression modulation, showing a 2.5-fold increase in overexpression lines and a 0.6-fold decrease in knockdown lines compared with respective controls. Western blotting also confirmed the corresponding increase and decrease in OGFR protein expression (Figure 4A).

Densitometric quantification of the Western blot signals demonstrated a 2.5 ± 0.3-fold increase in OGFR protein expression in the overexpression line compared with control, and a reduction to 0.58 ± 0.07 and 0.63 ± 0.05 in shOGFR1 and shOGFR2 cells, respectively (Appendix B(B1,B2)).

#### 3.4.1. Homologous Recombination Assay with OGFR-Overexpressing/Knockdown TFK-1 Cells via qPCR

To evaluate the effect of OGFR on HR activity, a qPCR-based HR assay was conducted. In OGFR-overexpressing TFK-1 cells, HR frequency decreased to 0.58 compared with control cells, whereas in shOGFR cells, it increased to 2.78 relative to shNTC controls. These findings suggest that OGFR negatively regulates HR activity in TFK-1 cells (Figure 4B).

#### 3.4.2. Quantitative Analysis of Cell-Cycle Distribution in OGFR-Overexpressing/Knockdown TFK-1 Cells via Flow Cytometry

Cell-cycle distribution was analyzed via flow cytometry. The proportion of cells in the G2/M phase slightly increased in OGFR-overexpressing cells compared with controls, whereas OGFR knockdown exerted negligible little effect on cell-cycle progression. These results suggest that OGFR overexpression is associated with a modest accumulation of cells in the G2/M phase (Figure 4C).

#### 3.4.3. Quantitative Analysis of Cell Death in OGFR-Overexpressing/Knockdown TFK-1 Cells with NIR Treatment via Flow Cytometry

Apoptosis was evaluated via flow cytometry. OGFR overexpression increased the proportion of apoptotic cells, particularly in combination with NIR treatment, whereas OGFR knockdown reduced basal apoptosis and alleviated the pro-apoptotic effect of NIR. These findings suggest that OGFR promotes apoptosis and enhances NIR-induced cytotoxicity in TFK-1 cells (Figure 4D).

#### 3.4.4. Quantitative Evaluation of Cell Proliferation in OGFR-Overexpressing/Knockdown TFK-1 Cells with NIR Treatment via Spectrophotometry

To evaluate the effect of OGFR on cell proliferation under NIR treatment, cell viability was measured daily from days 0 to 4 via CCK-8 assay, with values normalized to day 0. OGFR overexpression markedly suppressed cell proliferation, particularly in the presence of NIR, whereas OGFR knockdown had negligible effect compared with shNTC controls. These findings suggest that OGFR enhances the antiproliferative effect of NIR in TFK-1 cells (Figure 4E).

#### 3.4.5. Quantitative Evaluation of Cell Survival in OGFR-Overexpressing/Knockdown TFK-1 Cells with NIR Treatment via Spectrophotometry

To assess the impact of OGFR expression on NIR sensitivity in TFK-1 cells, dose–response analyses were conducted and IC_50_ values were calculated. OGFR overexpression reduced the IC_50_ of NIR, indicating enhanced drug sensitivity, whereas OGFR knockdown increased the IC_50_, suggesting relative resistance. These results indicate that OGFR expression modulates cellular responsiveness to NIR (Figure 4F).

### 3.5. Whole-Genome Bisulfite Sequencing After DNMT3B Knockdown

To elucidate the mechanism of OGFR upregulation induced by DNMT3B knockdown, bisulfite sequencing was performed using genomic DNA extracted from TFK-1 cells following siDNMT3B-3 and siDNMT3B-10 transfection.

The analysis revealed only a limited number of significant DMRs (FDR < 0.05) shared between the two knockdown groups, rendering pathway enrichment analysis for HR- or cell-cycle–related genes difficult.

In the comparison of methylation levels within the CpG island of the OGFR promoter region on chromosome 20 (approximately 62.80 M bp), a slight reduction in methylation was observed in siDNMT3B-3 cells compared with siNTC controls, whereas no reduction was seen in siDNMT3B-10 cells.

**Figure 5:**
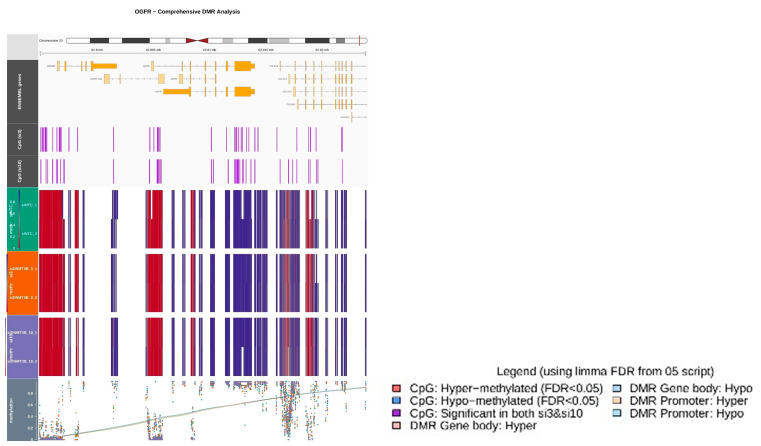
Whole-genome bisulfite sequencing after DNMT3B knockdown. Genome-wide DNA methylation profiling of TFK-1 cells after DNMT3B knockdown, analyzed using methylKit. The methylation status of the OGFR promoter region (chromosome 20, ~62.8 Mb) is presented.

These findings suggest that OGFR upregulation after DNMT3B knockdown is unlikely to be inducted by demethylation of its promoter region but rather by transcriptional regulatory mechanisms associated with DNMT3B suppression (Figure 5).

## 4. Discussion

This study demonstrated that DNMT inhibition markedly enhanced the antitumor effect of the PARP inhibitor NIR in PARP inhibitor-resistant BTC cell lines. This synergistic interaction was consistently reproduced by DNMT3B knockdown, providing compelling evidence that epigenetic modulation can sensitize BTC cells to PARP inhibitors and represent a novel therapeutic strategy for BTCs [17,18].

AZA monotherapy reduced HR frequency and, when combined with NIR, significantly decreased the IC_50_ value while enhancing apoptosis. These findings are consistent with previous reports regarding other tumor types, such as non-small-cell lung cancer and hematologic malignancies, in which DNA methylation inhibitors induced a “BRCAness” phenotype and enhanced the efficacy of PARP inhibitors [26].

DNMT3B knockdown phenotypically mimicked the effects of AZA, supporting a causal relationship between DNMT3B suppression, HR impairment, and increased sensitivity to PARP inhibitors. Among the DNMT family members, DNMT3B knockdown induced the greatest reduction in HR activity, increased apoptosis after NIR exposure, and markedly decreased the IC_50_ of NIR, whereas DNMT1 knockdown exerted only minimal effects. These findings are consistent with previous reports indicating that DNMT3B is a key regulator of chromatin structure and DNA repair capacity [27,28]. In addition to its canonical methyltransferase activity, DNMT3B also acts as a transcriptional co-regulator by interacting with chromatin-modifying complexes and transcription factors [29]. Therefore, the observed upregulation of the OGFR after DNMT3B knockdown is likely mediated by changes in DNMT3B-dependent transcriptional regulation rather than direct promoter demethylation.

Transcriptomic analysis identified OGFR as a commonly upregulated gene after DNMT3B knockdown. Functional assays revealed that OGFR overexpression suppressed HR activity, enhanced apoptosis, and increased sensitivity to NIR, whereas OGFR knockdown exerted opposite effects. These results suggest that OGFR acts as a novel regulator of HR repair and PARP inhibitor sensitivity in BTC. Although OGFR has been traditionally recognized as a G0/G1 checkpoint regulator, our findings indicated a mild increase in the G2/M population, suggesting a potential role for OGFR in modulating the DNA damage response or cell-cycle control in a context-dependent manner [30,31].

To address the reviewer’s concern regarding the mechanistic interpretation of OGFR, recent studies have expanded the functional scope of the OGF–OGFR axis beyond the classical growth-inhibitory pathway initially described in early reports. Somatic mutations of OGFR identified in human cancers have been shown to disrupt nuclear trafficking of the receptor and attenuate the growth-suppressive response to OGF, indicating that alterations in OGFR structure directly influence cancer cell proliferation and therapeutic sensitivity [32]. Furthermore, recent studies highlight that, in addition to classical opioid receptors, the OGF–OGFR signaling axis participates in broader tumor-associated processes, including modulation of inflammatory signaling, microenvironmental interactions, and responses to cytotoxic stress [33,34]. These updated findings suggest that OGFR exerts context-dependent regulatory effects that extend beyond simple G0/G1 checkpoint control, supporting a more complex model in which OGFR integrates multiple signaling cues relevant to cancer biology.

Whole-genome bisulfite sequencing revealed no significant CpG methylation changes in the OGFR locus. Specifically, analysis of the CpG island within the OGFR promoter region (chromosome 20, approximately 62.8 Mb) revealed only a slight decrease in methylation in siDNMT3B-3 cells compared with siNTC controls, whereas no reduction was observed in siDNMT3B-10 cells. The overall number of significant DMRs was limited (FDR < 0.05), precluding meaningful pathway enrichment analysis of HR- or cell-cycle-related genes. These findings suggest that OGFR upregulation after DNMT3B knockdown is unlikely to result from direct promoter demethylation but rather from transcriptional regulatory alterations associated with DNMT3B loss.

From a clinical perspective, these findings have significant implications. PARP inhibitors have shown clinical benefit in patients with BTC harboring HR repair-related gene mutations; however, pathogenic BRCA1/2 mutations are rare, limiting the eligible patient population [35]. Our results indicate that DNMT3B inhibition and OGFR upregulation converge to suppress HR activity, potentially expanding the therapeutic window of PARP inhibitors to include BTCs without germline HRR mutations. This strategy represents a form of synthetic lethality, wherein epigenetic modulation produces exploitable vulnerabilities in DNA damage repair pathways.

However, this study has several limitations that need to be acknowledged. First, all experiments were conducted in vitro using two BTC cell lines, which do not fully recapitulate the tumor microenvironment in vivo. Therefore, validation in xenograft or patient-derived models is warranted. Second, this study did not calculate the drug combination index, because we did not perform dose–response assays for AZA monotherapy or experiments using varying AZA:NIR ratios. The present work was designed primarily to clarify the relationship between DNMT3B suppression and PARP inhibitor sensitivity, rather than to optimize combination dosing. Prior to conducting future in vivo studies of AZA–NIR co-administration, in vitro combination index analysis will be required to rigorously characterize the nature of the combination effect. Third, although OGFR was shown to regulate HR and PARP inhibitor sensitivity, the exact mechanism of its transcriptional regulation remains unclear. Owing to the lack of CpG methylation changes, OGFR expression is likely regulated indirectly through DNMT3B-associated transcriptional networks, a hypothesis that requires further investigation. Fourth, RNA sequencing yielded relatively few DEGs, precluding comprehensive pathway enrichment analysis and system-level understanding of DNMT3B knockdown or AZA-induced changes. Fifth, while DNMT3B knockdown phenocopied AZA treatment in terms of HR suppression and PARP inhibitor sensitization, AZA did not induce substantial OGFR upregulation, indicating partially distinct mechanisms. As AZA can affect multiple epigenetic regulators beyond DNMTs, additional studies are warranted to elucidate these differences [36,37]. Finally, this study did not evaluate the effects of the combination of DNMT inhibition and other DNA damage response–targeting agents. Further exploration of such synthetic lethal interactions may reveal additional therapeutic opportunities in BTC.

In summary, DNMT inhibition sensitized BTC cells to PARP inhibitors, and OGFR was identified as a novel regulator of HR and drug sensitivity. The absence of considerable CpG methylation changes, alongside evidence of the transcriptional co-regulatory function of DNMT3B, suggests that OGFR upregulation is mediated by noncanonical DNMT3B-dependent transcriptional mechanisms. These findings provide new mechanistic insights into BTC biology and establish a rationale for combining epigenetic and PARP inhibitor therapies to improve clinical outcomes.

Future studies should aim to validate the efficacy of DNMT inhibition in combination with PARP inhibitors in vivo, ideally using xenograft or patient-derived models that capture the clinical heterogeneity of BTC. Furthermore, evaluating OGFR as a predictive biomarker can help identify patients most likely to benefit from this combination strategy. Ultimately, clinical trials combining DNMT and PARP inhibitors will be necessary to determine whether this approach can expand therapeutic options and improve outcomes for patients with BTC.

## 5. Conclusions

DNMT3B inhibition sensitizes BTC cells to PARP inhibitors by disrupting HR repair. OGFR was identified as a novel regulator of HR and PARP inhibitor sensitivity, controlled via noncanonical DNMT3B-dependent transcriptional mechanisms that operate independently of CpG methylation. These findings provide new mechanistic insights into the epigenetic control of DNA repair and support the rationale for combining DNMT and PARP inhibitors as a promising therapeutic strategy for BTC beyond genetically HR-deficient cases.

## Data Availability

The data presented in this study are available on request from the corresponding author. The data are not publicly available due to institutional data policy.

## Abbreviations

The following abbreviations are used in this manuscript:

AZA	5-Azacitidine
BTC	Biliary tract cancer
CCK-8	Cell Counting Kit-8
DEG	Differentially expressed gene
DNMT	DNA methyltransferase
DMR	Differentially methylated region
FDR	False discovery rate
FGFR	Fibroblast growth factor receptor
HR	Homologous recombination
HRR	Homologous recombination repair
IC50	Half maximal inhibitory concentration
NIR	Niraparib
OGFR	Opioid growth factor receptor
PARP	Poly(ADP-ribose) polymerase
PI	Propidium iodide
qPCR	Quantitative polymerase chain reaction
SEM	Standard error of the mean
siRNA	Small interfering RNA
shRNA	Short hairpin RNA

## Appendix A. Dose–Response Curves for Niraparib (NIR) in BTC Cell Lines

This figure presents the dose–response profiles of TFK-1 and RBE cells treated with increasing concentrations of niraparib (NIR; 0–50 µM). Both TFK-1 and RBE cells showed an initial decrease in cell number at approximately 5 µM NIR.

## Appendix B. Western Blot for OGFR Gene

B1 presents the original Western blot images used for Figure 4A, including all lanes and molecular weight markers. Blots were performed using anti-OGFR antibody (Sigma-Aldrich, NP_0313722) and β-actin as a loading control. Each experiment was repeated three times independently.

Band intensities were quantified using ImageJ with identical ROI sizes and background subtraction (rolling-ball algorithm). Protein expression levels were calculated as OGFR/β-actin ratios and normalized to each respective control group.
